# Vitamin D Ameliorates Fat Accumulation with AMPK/SIRT1 Activity in C2C12 Skeletal Muscle Cells

**DOI:** 10.3390/nu11112806

**Published:** 2019-11-17

**Authors:** Eugene Chang, Yangha Kim

**Affiliations:** Department of Nutritional Science and Food Management, Ewha Womans University, Seoul 03760, Korea

**Keywords:** adenosine monophosphate-activated protein kinase (AMPK), muscle, obesity, sirtulin 1 (SIRT1), vitamin D

## Abstract

Excessive fat accumulation has been considered as a major contributing factor for muscle mitochondrial dysfunction and its associated metabolic complications. The purpose of present study is to investigate a role of vitamin D in muscle fat accumulation and mitochondrial changes. In differentiated C2C12 muscle cells, palmitic acid (PA) was pretreated, followed by incubation with 1,25-dihyroxyvitamin D (1,25(OH)2D) for 24 h. PA led to a significant increment of triglyceride (TG) levels with increased lipid peroxidation and cellular damage, which were reversed by 1,25(OH)2D. The supplementation of 1,25(OH)2D significantly enhanced PA-decreased mtDNA levels as well as mRNA levels involved in mitochondrial biogenesis such as nuclear respiratory factor 1 (NRF1), peroxisome proliferative activated receptor gamma coactivator-1α (PGC-1α), and mitochondrial transcription factor A (Tfam) in C2C12 myotubes. Additionally, 1,25(OH)2D significantly increased ATP levels and gene expression related to mitochondrial function such as carnitine palmitoyltransferase 1 (CPT1), peroxisome proliferator-activated receptor α (PPARα), very long-chain acyl-CoA dehydrogenase (VLCAD), long-chain acyl-CoA dehydrogenase (LCAD), medium-chain acyl-CoA dehydrogenase (MCAD), uncoupling protein 2 (UCP2), and UCP3 and the vitamin D pathway including 25-dihydroxyvitamin D3 24-hydroxylase (CYP24) and 25-hydroxyvitamin D3 1-alpha-hydroxylase (CYP27) in PA-treated C2C12 myotubes. In addition to significant increment of sirtuin 1 (SIRT1) mRNA expression, increased activation of adenosine monophosphate-activated protein kinase (AMPK) and SIRT1 was found in 1,25(OH)2D-treated C2C12 muscle cells. Thus, we suggest that the observed protective effect of vitamin D on muscle fat accumulation and mitochondrial dysfunction in a positive manner via modulating AMPK/SIRT1 activation.

## 1. Introduction

A chronic positive energy surplus leads to obesity, which is characterized by enlarged adipose tissue [1,2]. Reduced fat storage capacity in adipose tissue, in association with increased body fat mass, causes ectopic fat accumulation in muscle, liver, pancreas, and heart, which is the link between obesity and its associated metabolic disease such as insulin resistance, type 2 diabetes mellitus, and cardiovascular disease [3,4,5]. Indeed, skeletal muscle from obese humans show increased intramuscular triglyceride levels [6,7,8] and decreased mitochondrial contents and function [9]. Therefore, it is important to regulate obesity-induced fat deposition and mitochondrial changes in skeletal muscle to prevent and/or treat obesity and its associated metabolic disorders. 

Several lines of evidence show the positive correlation between vitamin D deficiency and obesity [10,11]. There is a close association between low vitamin D status and reduced muscle mass, strength, and performance [12,13]. Previous studies illustrate that vitamin D plays a pivotal role in muscle cell proliferation and differentiation via vitamin D receptors (VDR), a family member of the steroid hormone receptor involved in vitamin D genomic regulation [14,15,16,17]. In addition to VDR, other vitamin D target genes, 25-dihydroxyvitamin D3 24-hydroxylase (CYP24) and 25-hydroxyvitamin D3 1-alpha-hydroxylase (CYP27) were expressed. CYP24 is the main degrading enzyme in the vitamin D turnover or signaling pathway by a hydroxylation at the 24 position. CYP27 leads to the extra-renal conversion of 25-hydroxyvitamin D (25(OH)D) to the most biologically active metabolite of vitamin D, 1,25-dihyroxyvitamin D (1,25(OH2)D) in muscle [17]. In addition, vitamin D improves muscle mitochondrial activity and functional capacity [18,19]. However, the relationship between vitamin D and the increment of intramyocellular lipid contents has been reported in clinical and basic studies [20,21,22]. The paradoxical effect that vitamin D enhances muscle function and that vitamin D increases the intramuscular fat levels, a risk factor for obesity and obesity-related complication, has not yet been fully explored. Moreover, it has never been investigated how vitamin D influences obesity-associated muscle fat deposition and mitochondrial changes. 

Previous studies demonstrate that increased muscle mitochondrial biogenesis and improved mitochondrial dysfunction in skeletal muscle are relevant to adenosine monophosphate-activated protein kinase (AMPK) and sirtuin 1 (SIRT1), a nicotinamide adenine dinucleotide (NAD)-dependent protein deacetylase [23,24]. A close association between reduced AMPK activity and obesity and its-associated metabolic dysfunctions has been documented in the skeletal muscle of obese humans [25,26]. In addition, SIRT1 plays an essential role in AMPK activation [27]. As main regulators of muscle fiber oxidative capacity and mitochondrial biogenesis, AMPK and SIRT1 influence the activation or transcripts of peroxisome proliferative activated receptor gamma coactivator-1α (PGC-1α) and nuclear respiratory factor 1 (NRF1) [28,29,30]. Due to the critical roles of AMPK and SIRT1 in muscle mitochondrial changes induced by obesity, they might be potential targets to prevent and/or treat obesity and obesity-related metabolic dysfunction. In the current study, the effect of vitamin D on lipid accumulation, mitochondrial changes, and activities of AMPK/SIRT1 were investigated in fatty acid-treated C2C12 muscle cells. 

## 2. Materials and Methods

### 2.1. Animals and Diets

A total of 23 Sprague-Dawley rats were purchased from Doo Yeol Biotech (Seoul, Korea). Rats were individually housed under a controlled environment; lighting (12 h light:12 h dark cycle), temperature (22 ± 2 °C), and humidity (55% ± 5%). To examine the role of vitamin D in muscle fat accumulation and mitochondrial changes, 4 week old rats were randomly assigned to three different diets as follows: (1) normal diet (NOR, 10% fat, 20% protein, and 70% carbohydrate diet with 1000 IU vitamin D/kg diet, Research Diets, Inc. (New Brunswick, NJ, USA)); (2) high fat diet (HF, 45% fat, 20% protein, and 30% carbohydrate diet with 1000 IU vitamin D/kg diet, Research Diets, Inc.); or (3) low vitamin D-contained HF diet (HF + LVD, 45% fat diet with 25 IU vitamin D/kg diet, Research Diets, Inc.). Body weight and food intake were measured twice a week for 12 weeks. After overnight fasting, rats were killed by CO_2_ followed by cardiac puncture. Skeletal muscle tissues were collected and immediately frozen in liquid nitrogen until further analysis. Animal housing procedures were approved by the Institutional Animal Care and Use Committee (IACUC) of the Ewha Womans University (IACUC No. 15-064).

### 2.2. Cell Culture and Treatment

Mouse C2C12 cell line was obtained from ATCC (American Type Culture Collection; Manassas, VA, USA). C2C12 myoblasts were cultured in a growth medium consisting of high glucose-contained Dulbecco’s Modified Eagle Medium (Gibco, Grand Island, NY, USA) supplemented with 10% fetal bovine serum (FBS; Gibco), 100 U/mL of penicillin (Gibco), and 100 mg/mL of streptomycin (Gibco) at 37 °C in 95% air and 5% CO_2_ atmosphere. Differentiation was induced by the transition from 10% FBS to 2% horse serum (Gibco) in the medium. On day 6 after inducing muscle differentiation, C2C12 myotubes were incubated with palmitic acid (PA, 0.5 mM; Sigma-Aldrich, St. Louis, MO, USA) for 24 h and treated with 1,25(OH)2D for an additional 24 h. For PA preparation, PA was complexed to fatty acid-free (>98%) bovine serum albumin (BSA; Sigma) at a 6:1 molar ratio. Sodium palmitate was dissolved in 150 mM NaCl solution at 70 °C water bath. Then, 1 mM PA solution was conjugated with 0.17 mM BSA solution in 150 mM NaCl solution by stirring for 1 h at 37 °C, filtered through a 0.2 μm filter (Sartorius, Gottingen, Germany), aliquot, and frozen at −20 °C. Absolute ethanol was used to dissolve 1,25(OH)2D to treat at a desired concentrations. 

### 2.3. Triglyceride Levels

As previously reported [31], dissected skeletal muscle tissue or harvest C2C12 muscle cells were homogenized in 5% Nonidet P-40 (NP-40) substitute (Sigma). Heated homogenates at 80–100 °C were cooled down to room temperature. A commercial triglyceride (TG) assay kit (Abcam, Cambridge, MA, USA) was used to quantify intramuscular or intramyocellular TG levels. Protein concentrations was measured by a bicinchoninic acid (BCA) protein assay kit (Thermo Scientific, Waltham, MA, USA). The TG concentrations were normalized to their respective protein concentrations.

### 2.4. Lipid Peroxidation

To determine levels of lipid peroxidation, malondialdehyde (MDA) contents were measured using a commercially available kit (Abcam). A lysis buffer with butylated hydroxytoluene was used to prevent peroxidation in the homogenates of C2C12 myotubes or skeletal muscle tissues. Homogenates were centrifuged at 13,000× *g* for 10 min at 4 °C. Free MDA present in the sample reacts with thiobarbituric acid (TBA) and generates an MDA-TBA adduct, which can be quantified at 532 nm. Lipid peroxidation was presented as the fold difference compared to NOR group or C2C12 control cells.

### 2.5. Lactate Dehydrogenase (LDH)

In the skeletal muscle from rats and C2C12 myotubes, lactate dehydrogenase (LDH) levels were determined using a colorimetric assay kit (Abcam). Nicotinamide adenine dinucleotide (NAD) to NADH was reduced by LDH, which was then calorimetrically quantified at 450 nm. LDH levels were normalized to total protein concentration, as determined by a BCA protein assay kit (Thermo Scientific).

### 2.6. Measurement of ATP Levels

Total ATP concentrations were quantitatively determined by an ATP assay kit (Abcam). In this assay, the product by glycerol phosphorylation was quantified at a wavelength of 570 nm. C2C12 myotubes were homogenized in 100 uL ATP assay buffer by pipetting up and down a few times. The homogenates were centrifuged at 13,000× *g* for 5 min at 4 °C to remove any insoluble materials. Intramyocellular ATP contents were normalized to their respective protein concentrations (Thermo Scientific).

### 2.7. RNA Isolation, Reverse Transcription, and Quantitative Real-Time Polymerase Chain Reaction (qRT-PCR)

Total RNA was isolated from muscle tissues and C2C12 muscle cells using an RNeasy Mini Kit (Qiagen, Valencia, CA, USA). A total of 1 μg of RNA was subsequently reverse-transcribed to cDNA using a MMLV reverse transcriptase kit (Bioneer, Daejeon, Korea) following the manufacturer’s instructions and the reaction was performed at 37 °C for 60 min by incubation at 95 °C for 5 min with the use of GeneAMP^®^ PCR system 2700 (Applied Biosystems, Foster City, CA, USA). The polymerase chain reaction (PCR) was performed using an AccuPower^®^ 2X GreenStar™ qPCR Master Mix (Bioneer) and a fluorometric thermal cycler (Corbett Research, NSW, Australia). The conditions used were as follows: pre-denaturation at 95 °C for 10 min, followed by 40 cycles of denaturation (95 °C, 15 s), annealing (60 °C, 20 s), and extension (72 °C, 20 s). Primers used are shown in Supplementary Tables S1 and S2. Results were relatively quantified using the ΔΔCt method and expressed as the fold change compared to NOR group or C2C12 vehicle control [32]. 

### 2.8. Transmission Electron Microscopy (TEM)

Fixation was carried out using 2% glutaraldehyde plus paraformaldehyde in a 0.1 M phosphate buffer (pH 7.4). After 2 h glutaraldehyde fixation at room temperature, fixed skeletal muscle tissues and myotubes were treated with 1% osmium tetroxide in 0.1 M PBS solution for 1 h (pH 7.4), dehydrated in gradually increasing concentrations of ethanol, and embedded in epoxy resin. Samples were then cut, stained, and examined using a transmission electron microscope (TEM) (Hitachi, Japan).

### 2.9. Mitochondrial DNA (mtDNA) Contents

Total DNA from muscle tissues and C2C12 muscle cells was isolated using a genomic DNA extraction kit (Bioneer), according to the manufacturer’s instruction. As described previously [33], mtDNA levels were determined using qRT-PCR by measuring the mitochondrial gene (*COX1*, subunit 1 of cytochrome oxidase) versus the nuclear gene (*GAPDH*, glyceraldehyde 3-phosphate dehydrogenase).

### 2.10. SIRT1 Activity

A SIRT1 activity assay kit from Abcam was used to determine SIRT1 activity. In brief, nuclear fractions from C2C12 myotubes or skeletal muscle were extracted. Nuclear SIRT1 activity was measured at 340 nm/460 nm in a combination with NAD and fluoro-substrate peptides and normalized to protein levels detected by a BCA protein assay kit (Thermo Scientific).

### 2.11. AMPK Activity

A semi-quantitative immunoassay method was employed to determine AMPK activation by an AMPK kinase assay kit (MBL International Co., Woburn, MA, USA). Muscle tissues and C2C12 muscle cells were homogenized in a lysis buffer including protein and phosphatase inhibitors (Sigma). After centrifugation at 13,000× *g* for 20 min at 4 °C, insoluble materials were removed. AMPK activity was measured in the aqueous part at a wavelength of 450 nm using a microplate reader (Varioskan Flash, Thermo Scientific), normalized to their respective protein concentrations as determined by a BCA protein assay (Thermo Scientific), and expressed as the fold difference to values from NOR group or C2C12 vehicle control.

### 2.12. Statistical Analysis

All statistical analyses were performed using SPSS Statistics 20 (SPSS, Inc., Chicago, IL, USA). Data are expressed as mean ± standard error of the mean (SEM). One-way analysis of variance (ANOVA) followed by Student–Newman–Keuls multiple comparison post hoc test or one-tailed Student’s *t*-test were used. Statistical significance was defined as *p* < 0.05.

## 3. Results

### 3.1. Effects of Vitamin D-Inadequate Diet on Body Weight Gain and Weight of Skeletal Muscle

**Figure 1:**
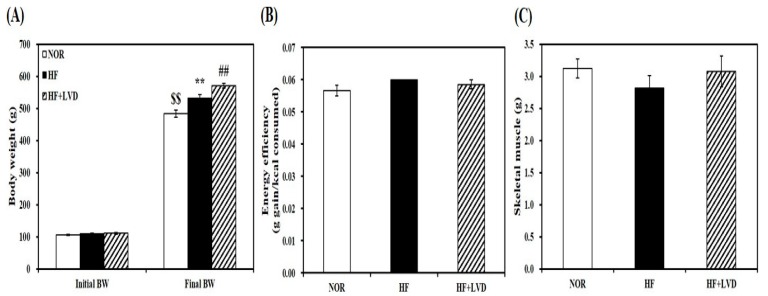
Vitamin D-inadequate diet induces obesity in HF-fed obese rats. Initial and final body weight (**A**), energy efficiency (**B**), and weight of skeletal muscle (**C**). Data is expressed as mean ± SEM (*n* = 7–9 per group). $$ *p* < 0.01 compared to initial body weight. ** *p* < 0.01 compared to NOR. ## *p* < 0.01 compared to HF. NOR, 10% fat diet with 1000 IU vitamin D; HF, 45% fat diet with 1000 IU vitamin D; HF + LVD, 45% fat diet containing 25 IU vitamin D.

The effect of vitamin D status on muscle fat accumulation was examined by using HF diet with two vitamin D levels. For dietary vitamin D levels, 25 and 1000 IU vitamin D/kg of diet were chosen to achieve vitamin D inadequacy or adequacy without adverse effects [34,35,36]. 12-week supplementation of 25 IU vitamin D/kg of diet (HF + VD) led to vitamin D insufficiency as featured by a blood level of 25-hydroxyvitamin D (25(OH)D) less than 30 ng/mL [37,38]. Rats fed on either NOR or HF containing 1000 IU vitamin D/kg of diet, the NRC vitamin D requirement [39] had circulating 25(OH)D concentrations above 30 ng/mL which is regarded as an adequate level in humans [37,38]. HF diet-increased body weight was aggregated by vitamin D-insufficient diet without changing energy efficiency (Figure 1A,B). In addition, average weight of skeletal muscle was not statistically different according to vitamin D levels within the diet (Figure 1C). 

### 3.2. Vitamin D Insufficiency Increases Intramuscular Fat Deposition, Lipid Peroxidation, and Tissue Damage

**Figure 2:**
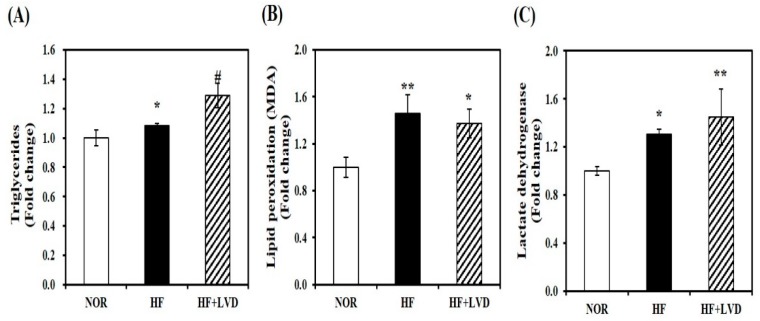
Influence of vitamin D insufficiency on muscle fat deposition, lipid peroxidation, and tissue damage. Muscle triglyceride concentrations (**A**), malondialdehyde (MDA) contents (**B**), and lactate dehydrogenase (LDH) levels (**C**) were normalized to their respective protein concentrations and expressed as the fold change compared to NOR. Data are expressed as mean ± SEM (*n* = 7–9). * *p* < 0.05; ** *p* < 0.01 compared to NOR. # *p* < 0.05 compared to HF. NOR, 10% fat diet with 1000 IU vitamin D; HF, 45% fat diet with 1000 IU vitamin D; HF + LVD, 45% fat diet containing 25 IU vitamin D.

To examine the influence of vitamin D status on obesity-associated muscle fat accumulation, we measured TG levels in the skeletal muscle tissues. Vitamin D insufficiency (HF + LVD) significantly increased muscle TG levels by about 19%, compared to the HF group (Figure 2A). Next, a marker of oxidative stress and tissue damage was detected by MDA or LDH levels to investigate the effect of vitamin D deficiency on oxidative stress and damage within the skeletal muscle. HF diet significantly increased muscle lipid peroxidation or tissue damage by 30% or 23%, respectively, which were not significantly aggravated by vitamin D insufficiency (Figure 2B,C).

### 3.3. Influence of Vitamin D-Inadequate Diet on Muscle Mitochondrial Morphology and mtDNA Contents

**Figure 3:**
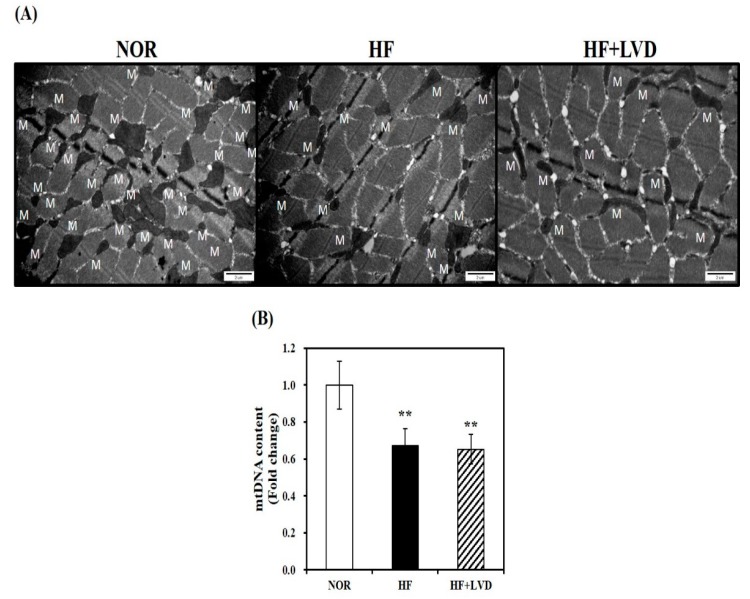
Mitochondrial morphology and mitochondrial DNA (mtDNA) contents in skeletal muscle fed with either vitamin D-adequate or insufficient diet for 12 weeks. (**A**) Electron microscopy of muscle (magnification of 20,000; scale bars = 2 μm). M indicates the position of mitochondria. The mtDNA contents were quantified by qRT-PCR (**B**). Values are expressed as mean ± SEM (*n* = 7–9 per group). ** *p* < 0.01 compared to NOR. NOR, 10% fat diet with 1000 IU vitamin D; HF, 45% fat diet with 1000 IU vitamin D; HF + LVD, 45% fat diet containing 25 IU vitamin D.

A TEM was used to observe morphological changes in muscle mitochondria. We observed a smaller size and decreased number of skeletal muscle mitochondria in the HF group, compared to the NOR group, however, no further obvious morphological changes were observed in the vitamin D deficient group (Figure 3A). Next, we analyzed mtDNA levels, an indicator of mitochondrial mass which is associated with the increase of mitochondrial number and size by regulating a major transcription factor related to mitochondrial biogenesis. As shown in Figure 3B, the HF group had lower mtDNA contents than the NOR group (*p* < 0.05). However, no further reduction of mtDNA levels was found by vitamin D insufficiency (Figure 3B). 

### 3.4. Effect of Vitamin D Inadequacy on Muscle mRNA Expression Involved in Mitochondrial Biogenesis and Function

**Figure 4:**
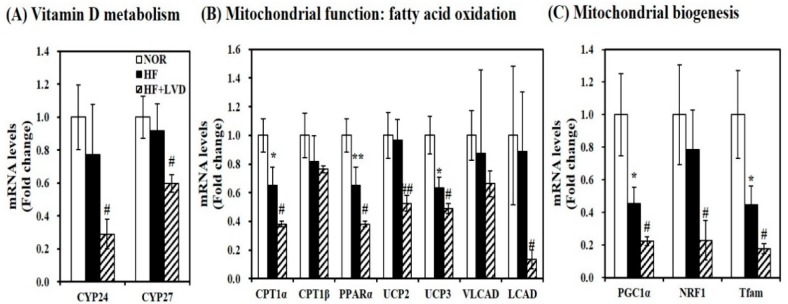
Vitamin D insufficiency decreases muscle mRNA levels related to vitamin D metabolism and mitochondrial biogenesis and function in HF-induced obese rats. mRNA levels were determined by qRT-PCR, normalized for all samples to β-actin, and expressed as the fold change compared to the NOR group. (**A**) vitamin D responsive genes; (**B**) mitochondrial function; (**C**) mitochondrial biogenesis). Results are expressed as mean ± SEM (*n* = 7–9 per group). * *p* < 0.05; ** *p* < 0.01 compared to NOR. # *p* < 0.05; ## *p* < 0.01 compared to HF. NOR, 10% fat diet with 1000 IU vitamin D; HF, 45% fat diet with 1000 IU vitamin D; HF + LVD, 45% fat diet containing 25 IU vitamin D.

Although vitamin D insufficiency aggravates muscle fat infiltration, no further significant changes in lipid peroxidation, intramuscular damage, and mtDNA levels were found in obese rats. Given the close association between vitamin D and muscle fat deposition, next, we investigated whether vitamin D status influences muscle mRNA levels related to vitamin D metabolism. Low vitamin D levels in the HF diet significantly decreased muscle CYP24 and CYP27 mRNA expression by 62.5% and 34.8%, respectively, compared to the HF diet (Figure 4A).

To investigate the influence of vitamin D on muscle mitochondrial dynamics, gene expression related to mitochondrial biogenesis and function was measured using qRT-PCR. As shown in Figure 4B, vitamin D inadequacy significantly suppressed muscle gene expression involved in fatty acid oxidation such as carnitine palmitoyltransferase 1α (CPT1α), peroxisome proliferator-activated receptor α (PPARα), very long-chain acyl-CoA dehydrogenase (VLCAD), and long-chain acyl-CoA dehydrogenase (LCAD) by 41.5%, 50.7%, 41.5%, 24.4%, and 84.9%, respectively (*p* < 0.05). Additionally, the inhibitory effect of vitamin D inadequacy on muscle uncoupling protein 2 (UCP2) and UCP3 mRNA levels were found by 45.6% and 22.7% in HF-induced obese rats (Figure 4B). Furthermore, vitamin D-inadequate HF diet significantly decreased muscle transcripts related to mitochondrial biogenesis such as PGC-1α, NRF1, and mitochondrial transcription factor A (Tfam) gene expression were by 50.7%, 70.9%, and 60.5% compared to the HF diet (Figure 4C). 

### 3.5. Vitamin D Inadequacy Decreases Muscle SIRT1 and AMPK Activities

**Figure 5:**
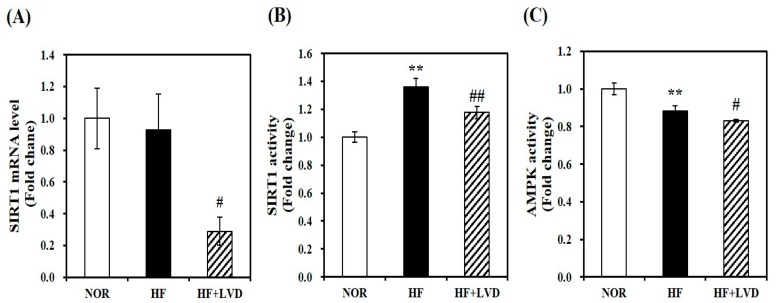
The effect of vitamin D inadequacy on sirtuin 1 (SIRT1) gene expression and adenosine monophosphate-activated protein kinase (AMPK)/SIRT1 activation in muscle tissues. SIRT1 mRNA expression was determined by qRT-PCR, normalized for all samples to β-actin, and expressed as the fold change compared to NOR group (**A**). Measurement of SIRT1 activity (**B**) and AMPK activity (**C**) was carried out using commercial assay kits, normalized to protein concentration, and expressed as the fold change to the NOR group. Results are expressed as mean ± SEM (*n* = 7–9 per group). ** *p* < 0.01 compared to NOR. # *p* < 0.05; ## *p* < 0.01 compared to HF. NOR, 10% fat diet with 1000 IU vitamin D; HF, 45% fat diet with 1000 IU vitamin D; HF + LVD, 45% fat diet containing 25 IU vitamin D.

The influence of vitamin D status on activities of SIRT1 and AMPK involved in obesity-induced muscle fat accumulation through muscle mitochondrial changes has never been evaluated. Thus, we determined whether vitamin D insufficiency suppresses mRNA transcript and activities of SIRT1 and AMPK in skeletal muscle tissues from HF-induced obese rats. Muscle SIRT1 mRNA expression was significantly inhibited by 68.9% in vitamin D-insufficient rats (*p* < 0.05) (Figure 5A). Consistent with SIRT1 transcripts, muscle SIRT1 activity was significantly decreased by 13.4% in obese rats with vitamin D insufficiency, compared to vitamin D adequate obese animals (Figure 5B). As shown in Figure 5C, HF diet significantly decreased muscle AMPK activity compared to the NOR group. There was additional decrement of muscle AMPK activity in vitamin D-insufficient obese rats (*p* < 0.05). 

### 3.6. In Vitro Effects of Vitamin D on Palmitate-Increased Lipid Accumulation, Lipid Peroxidation, and Cellular Damage in C2C12 Muscle Cells

In an animal study with two vitamin D levels in the HF diet, vitamin D insufficiency significantly aggravated HF-increased body weight and muscle fat accumulation with decreased mRNA levels involved in vitamin D metabolism and muscle function and biogenesis. However, no further changes in mtDNA levels, lipid peroxidation, and tissue damage were indicated by vitamin D insufficiency. To explain the direct effect of vitamin D on ectopic fat accumulation and aberrant mitochondrial changes in obese skeletal muscle, an in vitro cell culture supplemented with 1,25(OH)2D, the most biologically active form, was carried out. Differentiated C2C12 cells were pretreated with PA (0.5 mM, 24 h) followed by incubation with 1,25(OH)2D for 24 h. 

**Figure 6:**
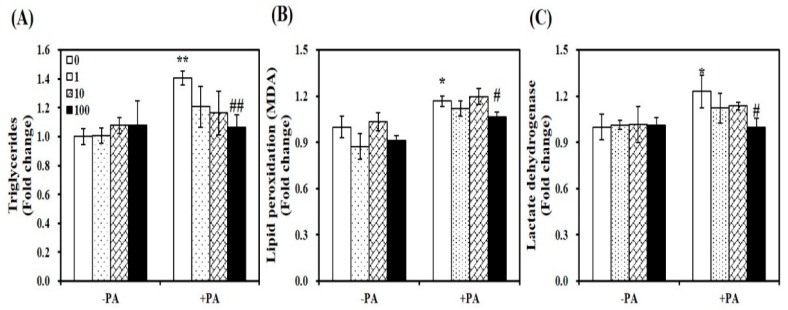
Inhibitory effect of 1,25-dihydroxyvitamin D (1,25(OH)2D) on lipid accumulation, lipid peroxidation, and cellular damage. C2C12 myotubes were pretreated with palmitic acid (0.5 mM, 24 h) and then incubated with 1,25(OH)2D (0, 1, 10, or 100 nM, 24 h). Intramyocellular triglyceride concentrations (**A**), malondialdehyde (MDA) contents (**B**), and lactate dehydrogenase (LDH) levels (**C**) were expressed as the fold change compared to the vehicle control. Results are expressed as mean ± SEM. Experiments represent at least two or three independent experiments (*n* = 6–10 per group). * *p* < 0.05; ** *p* < 0.01 compared to vehicle control. # *p* < 0.05; ## *p* < 0.01 compared to PA control.

As shown in Figure 6A, 1,25(OH)2D treatment significantly dose-dependently suppressed lipid infiltration in PA-loaded myotubes. At a dose of 100 nM for 24 h, 1,25(OH)2D supplementation led to an approximately 24% significant decrease of TG concentrations, compared to PA-incubated muscle cells (*p* < 0.01). PA-induced increments in intramyocellular lipid peroxidation and damage, as determined by levels of lipid peroxidation (MDA) and LDH were significantly decreased by 1,25(OH)2D treatment (100 nM, 24 h) (Figure 6B,C). Thus, vitamin D supplementation has favorable effects on PA-induced fat accumulation, lipid peroxidation, and intracellular damage in C2C12 myotubes. 

### 3.7. Influence of 1,25(OH)2D on PA-Induced Mitochondrial Changes and ATP Levels in C2C12 Myotubes

**Figure 7:**
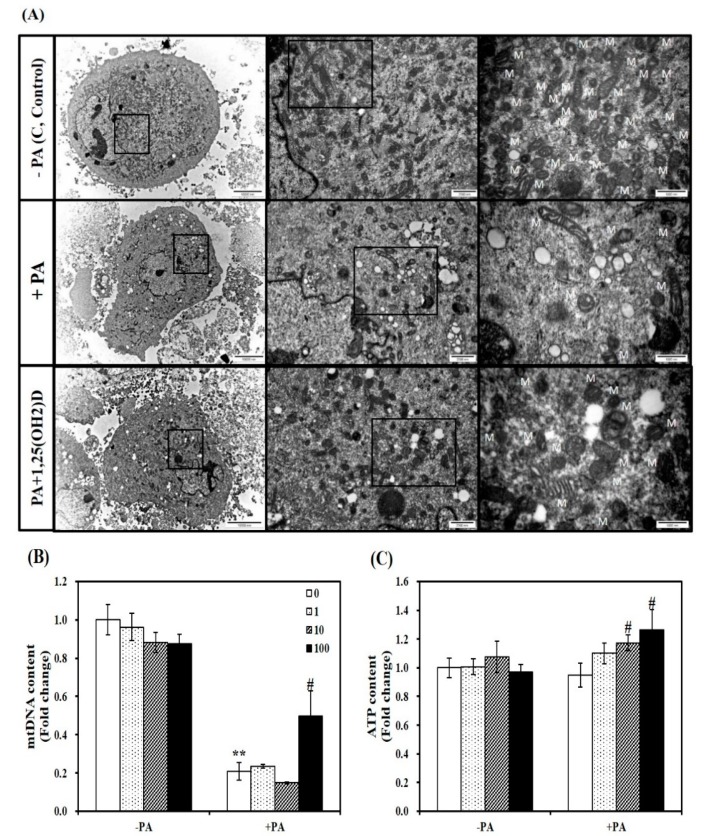
Effect of 1,25-dihydroxyvitamin D (1,25(OH)2D) on fatty acid-induced changes in mitochondrial morphology, mitochondrial DNA (mtDNA) contents, and ATP levels in C2C12 muscle cells. (**A**) Transmission electron microscopy of myotubes (magnification of 7000, 20,000, and 50,000; scale bars = 10, 2, and 1 μm). M indicates the position of mitochondria. qRT-PCR was used to measure mtDNA transcripts (**B**). ATP concentrations were measured using a colorimetric ATP assay kit, normalized to their relative protein levels, and expressed as the fold change compared to the vehicle control (**C**). Values are expressed as mean ± SEM from at least two or three independent experiments (*n* = 6–8 per group). ** *p* < 0.01 compared to vehicle control. # *p* < 0.05 compared to PA control. C, vehicle-treated C2C12 muscle cells; PA, 0.5 mM palmitic acid-treated myotubes; PA + 1,25(OH)2D, pretreatment with 0.5 mM PA following 1,25(OH)2D incubation (100 nM, 24 h).

To demonstrate the influence of vitamin D supplementation on aberrant mitochondrial changes, we observed mitochondrial morphology by TEM. An increase in mitochondrial number and size were observed in 1,25(OH)2D-treated C2C12 muscle cells, compared to PA-incubated cells (Figure 7A). Consistent with this observation, 1,25(OH)2D (100 nM, 24 h) significantly increased mtDNA contents by 2.3-fold (*p* < 0.05, Figure 7B). Next, we investigated the influence of 1,25(OH)2D on ATP levels, which is correlated with mitochondrial number and size. The supplementation of 1,25(OH)2D to C2C12 myotubes significantly dose-dependently increased ATP contents compared to myotubes treated with PA with a significant increase at 10 nM 1,25(OH)2D (Figure 7C). Based on 1,25(OH)2D-increased levels of mtDNA and ATP, we suggest that increased vitamin D status might protect against PA-decreased muscle mitochondrial changes.

### 3.8. Effect of 1,25(OH)2D on mRNA Expression Related to Mitochondrial Biogenesis and Function in C2C12 Myotubes

**Figure 8:**
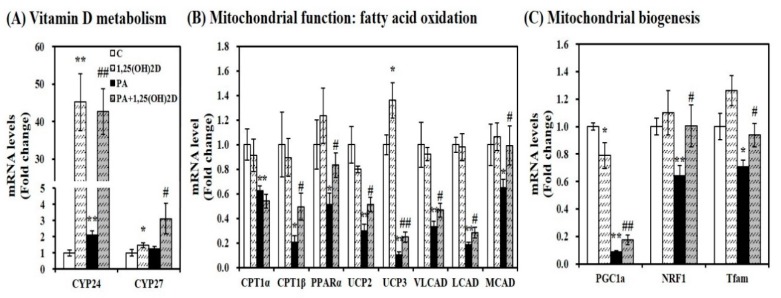
Influence of 1,25-dihydroxyvitamin D (1,25(OH)2D) on mRNA expression involved in vitamin D metabolism and mitochondrial biogenesis and function in C2C12 muscle cells. Gene expression was determined by RT-PCR and normalized for all samples to β-actin. (**A**) vitamin D responsive genes; (**B**) mitochondrial function; (**C**) mitochondrial biogenesis. Results represent mean ± SEM from two experiments (*n* = 6 per group). * *p* < 0.05; ** *p* < 0.01 compared to vehicle control. # *p* < 0.05; ## *p* < 0.01 compared to PA control. C, vehicle treated C2C12 muscle cells; PA, 0.5 mM palmitic acid-treated myotubes; PA + 1,25(OH)2D, pretreatment with 0.5 mM PA following 1,25(OH)2D incubation (100 nM, 24 h).

Given the close association between intramyocellular lipid accumulation and mitochondrial changes, we sought to determine gene expression involved in vitamin D metabolism and mitochondrial biogenesis and function. Regardless of the usage of PA, 1,25(OH)2D treatment (24 h, 100 nM) significantly enhanced mRNA expression related to 1,25(OH)2D responsive genes such as CYP24 and CYP27 in C2C12 myotubes (Figure 8A). In PA-loaded differentiated C2C12 myotubes, 1,25(OH)2D treatment (24 h, 100 nM) significantly increased mRNA expression involved in β-fatty oxidation such as CPT1β, PPARα, UCP2, UCP3, VLCAD, LCAD, and medium-chain acyl-CoA dehydrogenase (MCAD) by 2.38, 1.62, 1.73, 2.46, 1.40, 1.53, and 1.53-fold, respectively (Figure 8B). In 1,25(OH)2D-supplemented myotubes, mRNA levels of PGC-1α, NRF1, and Tfam involved in mitochondrial biogenesis were significantly was increased by 2.01, 1.56, and 1.32-fold, respectively, compared to PA-treated C2C12 muscle cells (Figure 8C). 

### 3.9. 1,25(OH)2D Promotes SIRT1 and AMPK Activation in Differentiated Muscle Cells

**Figure 9:**
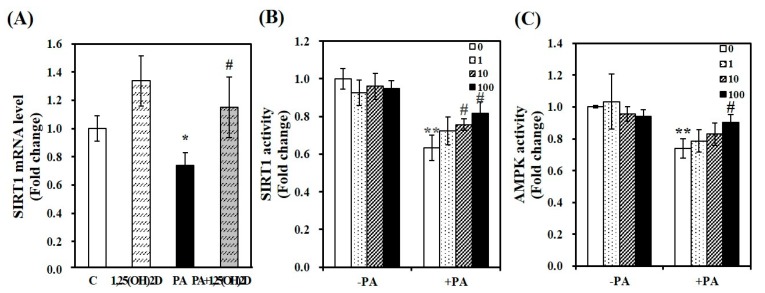
1,25-dihydroxyvitamin D (1,25(OH)2D) increases SIRT1 gene expression and AMPK/SIRT1 activation in C2C12 muscle cells. SIRT1 mRNA levels were determined by RT-PCR and normalized for all samples to β-actin (**A**). SIRT1 activity (**B**) and AMPK activity (**C**) were analyzed by a fluorometric SIRT1 activity assay kit or an AMPK kinase kit, normalized to their relative protein concentrations, and expressed as the fold change compared to the vehicle control. The value of each bar represents mean ± SEM. Experiments represent at least two or three independent experiments (*n* = 6–8 per group). * *p* < 0.05; ** *p* < 0.01 compared to vehicle control. # *p* < 0.05 compared to PA control. C, vehicle treated C2C12 muscle cells; PA, 0.5 mM palmitic acid-treated myotubes; PA + 1,25(OH)2D, pretreatment with 0.5 mM PA following 1,25(OH)2D incubation (100 nM, 24 h).

Next, we determined whether 1,25(OH)2D protects PA-increased intracellular lipid deposition and mitochondrial changes by modulating the activities of SIRT1 and AMPK. As shown Figure 9A, the supplementation of 1,25(OH)2D reversed PA-decreased SIRT1 mRNA level in C2C12 muscle cells. Additionally, an increment of SIRT1 activity was dose-dependently observed in 1,25(OH)2D-treated myotubes with a significant increase at 10 nM. Compared to PA-incubated cells, a 1.3-fold maximal SIRT1 activity was found in 100 nM 1,25(OH)2D-treated muscle cells (*p* < 0.05, Figure 9B). Next, we examined whether 1,25(OH)2D promotes AMPK activity. There was a further increasing effect of 1,25(OH)2D on AMPK activity in PA-loaded cells (Figure 9C). 

## 4. Discussion

Increased intramuscular triglyceride contents and decreased mitochondrial size and its function, and lipid oxidation, have been reported in the skeletal muscle from obese humans and rodents [6,7,8,9,40,41]. Vitamin D insufficiency has shown a close association with the prevalence of obesity [10,11,36]. However, the influence of vitamin D on obesity-induced fat accumulation and mitochondrial changes within the skeletal muscle has never been investigated. In the present study, we showed that vitamin D-inadequate diet significantly exacerbated obesity-induced intramuscular fat accumulation in rats fed with a HF diet and suppressed AMPK/SIRT1 activities. In contrast to the influence of vitamin D insufficiency on skeletal muscle tissues from obese rats, 1,25(OH)2D supplementation significantly reduced fat deposition, lipid peroxidation, intramyocellular damage, and increased mitochondrial contents and ATP levels and the activities of SIRT1 and AMPK in PA-incubated C2C12 myotubes. It suggests that vitamin D supplementation might improve obesity-increased intramyocellular fat deposition and its associated muscle mitochondrial changes, concurrently with an increase in AMPK/SIRT1 activity. 

Previous studies demonstrate low vitamin D status has been associated with obesity in human subjects and rodents [10,11,36]. In addition, a positive relationship between vitamin D deficiency and the incidence of falls and hip fractures have been found in the elderly, which is reversible by vitamin D supplementation [42,43,44]. Based upon the fact that fat accumulation in muscle tissue, which is an independent risk factor for obesity-related diseases, has been increased by obesity, there is a conflict that vitamin D increases muscle fat mass and instead vitamin D has a positive effect on obesity [20,21,22]. However, the influence of vitamin D on muscle ectopic fat accumulation has never been fully determined during the development of obesity. Therefore, we first delineated the favorable effects of vitamin D on HF-increased fat accumulation within the skeletal muscle of obese rats. The 25 IU vitamin D/kg diet yielded vitamin insufficiency as defined by less than 30 ng/mL blood 25(OH)D level [37,38]. Moreover, our previous study demonstrates the time course of body weight gain and food intake for 12 weeks. The 25 IU vitamin D/kg HF diet significantly increased body weight gain and food intake with adipose tissue expansion and inflammation [36]. In the present study, we delineated the role of vitamin D in ectopic fat accumulation and mitochondrial changes in muscle tissue. Indeed, the significant increment of muscle triglyceride levels were found in HF-induced obese rats with vitamin D insufficiency, compared to obese animals in a vitamin D-adequate condition. However, further research is warranted to investigate whether vitamin D insufficiency-induced change of food intake is directly associated with decreased ectopic fat infiltration in skeletal muscle. To investigate the direct effect of vitamin D on muscle fat accumulation in a positive condition of energy/fat surplus, in the current study, an in vitro cell culture was carried out. Similar to the in vivo animal study showing an inverse association between vitamin D status and muscle fat deposition, the supplementation of 1,25(OH)2D significantly reduced PA-induced intramyocellular fat deposition. Both in vivo and in vitro results demonstrate the favorable effect of vitamin D on fat accumulation within the skeletal muscle.

Increased fat infiltration within the skeletal muscle has been linked to a decrease of muscle mitochondrial function and size, which is associated with increased susceptibility of obesity and its related health complications [8,9,40,45,46]. In the present study, HF diet-induced obese rats had less mtDNA contents in skeletal muscle. However, an additional effect of vitamin D insufficiency on HF-decreased muscle mtDNA levels were not found. The direct effect of vitamin D on obesity-induced muscle fat deposition and its relevant mitochondrial changes might be difficult due to above 30 ng/mL of circulating 25(OH)D in obese rats. To delineate the effect of vitamin D on muscle fat accumulation and mitochondrial dynamics, in the current study, the supplementation of 1,25(OH)2D, the most active metabolite of vitamin D to differentiated muscle cells was executed. In in vitro cultured C2C12 myotubes, 1,25(OH)2D treatment displayed enlarged mitochondrial size, as observed by TEM and significantly increased mtDNA contents. Contrary to the results between in vitro muscle cells supplemented with 1,25(OH)2D, a direct effect of muscle mitochondrial changes such as levels of mtDNA, lipid peroxidation, and intramuscular damage could not be observed in obese rats with vitamin D inadequacy. Therefore, further studies are needed in vitamin D-deficient animal and/or cell culture models using VDR deletion or vitamin D-depletion methods, along with an in-depth analysis for measuring mitochondrial cross-sectional area and volume density.

With respect to the mechanisms regarding how vitamin D prevents intramuscular lipid accumulation, previous studies illustrate the link between vitamin D supplementation and muscle mitochondrial function [18,19]. The 1,25(OH)2D treatment significantly decreased lipid peroxidation and cellular death and the increment of ATP concentrations in PA-incubated myotubes. Several studies have shown that lipid fusion, fatty acid overload, or administration of HF diet to human subjects, rodents, or in vitro cultured muscle cells lead to impaired mitochondrial function, characterized by reduced ATP content, increased oxidative stress and apoptosis, and impaired oxidative capacity and oxidative phosphorylation [8,47,48,49,50]. It suggests that mitochondrial dysfunction might be closely associated with the pathophysiology of obesity-associated health outcomes [8,9,40,45,46]. Hence, any intervention capable of reducing lipid infiltration, oxidative stress, apoptosis, and augmenting oxidative capacity and phosphorylation might be of therapeutic importance. In our previous study, when compared to the HF diet, a further increment of fasting insulin concentrations about by 28.6% was found in vitamin D insufficient-HF diet [36]. Thus, we suggest that vitamin D-improved muscle fat infiltration and mitochondrial dysfunction might be in part related to beneficial effects of vitamin D on obesity and its related health complications. Still, it is necessary to investigate the mechanism by which vitamin D-induced intramuscular TG levels and mitochondrial changes influence obesity and obesity-related insulin resistance and type 2 diabetes by measuring whole-body expenditure, oxygen consumption, glucose tolerance test, insulin tolerance test, and hyperinsulinemic-euglycemic clamps.

Several transcription factors are involved in muscle mitochondrial biogenesis and function. In a condition of high intramyocellular lipid contents, a reduction of PGC1α has been reported [48,49]. PGC1α is a transcriptional coactivator for NRF1 and Tfam [51,52], which stimulates replication and transcription of mtDNA [53,54]. Given the association between coordinated expression of structural and regulatory genes and mtDNA, mtDNA content seems to be linked to mitochondrial mass. In addition, the interaction of PGC1α with PPARα stimulates the fatty acid oxidation-related gene, CPT1 [55]. Mitochondrial enzymes, VLCAD, LCAD, and MCAD are responsible for fatty acid β-oxidation [56]. Another mitochondrial membrane protein, muscle UCP2 and UCP3 are involved in weight maintenance by altering metabolic function such as thermogenesis and energy homeostasis [57]. In the present study, we demonstrate the role of vitamin D in muscle mitochondrial dynamics by measuring gene expression involved in mitochondrial biogenesis and function. In PA-treated C2C12 muscle cells, 1,25(OH)2D supplementation significantly increased gene expression involved in fatty acid oxidation and metabolic function such as CPT1, PPARα, VLCAD, LCAD, and MCAD as well as UCP2 and UCP3. In PA-loaded muscle cells, 1,25(OH)2D significantly upregulated PA-decreased mRNA levels of PGC1α, NRF1, and Tfam D. As reported in previous studies [17,20], CYP24 and CYP27 gene expression was increased by 1,25(OH)2D. Thus, it suggests that vitamin D-decreased ectopic fat infiltration might be associated with increased mRNA levels related to mitochondrial biogenesis and function together with vitamin D signaling in muscle. However, there was no direct and definite link between mtDNA contents and muscle mRNA transcripts involved in mitochondrial biogenesis and function in obese rats with vitamin D insufficiency. Low vitamin D-contained HF diet significantly aggravated HF-decreased muscle gene expression involved in vitamin D responsive genes including CYP24 and CYP27, fatty acid β-oxidation such as CPT1, PPARα, UCP2, UCP3, VLCAD, and LCAD and mitochondrial biogenesis including PGC1, NRF1, and Tfam without changing mtDNA copy number. Therefore, a further study to investigate the influence of vitamin D deficiency on muscle mitochondrial biogenesis in a way of using VDR genetic modification or vitamin D-depleted diet may be warranted. 

Emerging evidence demonstrates a critical role of two regulators, SIRT1 and AMPK [23,24]. AMPK activity increases muscle oxidative capacity by stimulating mitochondrial biogenesis, which is initiated by AMPK phosphorylation of transcription substrates such as PGC1α and NRF1, as well as fatty acid oxidation [28,58,59]. SIRT1 contributes to PGC1α deacetylation and its activation [29,30]. A line of evidence displays overlapped AMPK and SIRT1 signaling in enhancing muscle mitochondrial biogenesis and β-oxidation; SIRT1 activation is required for AMPK phosphorylation; however, AMPK activates SIRT1 by the increase of NAD levels and biosynthesis thereby influencing PGC1α deacetylation [27,60]. Given this close association between AMPK/SIRT1 activation and muscle mitochondrial biogenesis and function, AMPK/SIRT1 could be a critical target for the prevention/treatment of obesity and its related metabolic effects. In the current study, we revealed that vitamin D insufficiency significantly inhibited muscle AMPK/SIRT1 activation with significant reduction of SIRT1 mRNA expression in HF-induced obese rats. In addition, 1,25(OH)2D supplementation showed an increase in AMPK and SIRT1 activities and SIRT1 transcription levels. In the link between AMPK and SIRT1 activities, the NAD to NADH ratio was significantly increased by 1,25(OH)2D in PA-loaded myotubes (Figure S1). These data suggest that 1,25(OH)2D-decreased fat accumulation and mitochondrial dynamics might be related to enhanced AMPK/SIRT1 activation. 

Taken together, the observed effect of vitamin D on muscle fat accumulation and mRNA levels involved in mitochondrial biogenesis and function with AMPK/SIRT1 activation may demonstrate the beneficial effect of vitamin D on obesity and its associated metabolic disorders. 

## Supplementary Materials

The following are available online at https://www.mdpi.com/2072-6643/11/11/2806/s1, Figure S1: 1,25-dihydroxyvitamin D (1,25(OH)2D) increases the ratio of NAD to NADH, Table S1: Mouse primers used for quantitative real-time polymerase chain reaction (qRT-PCR), Table S2: Rat primers used for quantitative real-time polymerase chain reaction (qRT-PCR).
