# Supplementary material for: Vitamin D Ameliorates Fat Accumulation with AMPK/SIRT1 Activity in C2C12 Skeletal Muscle Cells
Source: Nutrients. 2019 Nov 17;11(11):2806. doi: 10.3390/nu11112806 (PMC6893473; doi:10.3390/nu11112806)
Supplement: Supplementary file 1 [file nutrients-11-02806-s001.pdf]

nutrients MDPI

Table S1. Mouse primers used for quantitative real-time polymerase chain reaction (qRT-PCR)

Gene	GeneBank no.	Forward sequence (5′-3′)	Reverse sequence (5′-3′)	Product size (bp)
β-actin	NM_007393.5	GGACCTGACAGACTACCTCA	GTTGCCAATAGTGATGACCT	208
CPT1α	NM_013495.2	GTGTTGGAGGTGACAGACTT	CACTTTCTCTTTCCACAAGG	100
CPT1β	NM_009948.2	CTCCGAAAAGCACCAAAACA	CTCCAGCACCCAGATGATTG	221
CYP24	NM_009996.4	AGTGAACCTGTGGAGATGCTG	AGTTGTCCCATGCTCTGGTC	201
CYP27	NM_010009.2	GTGGGCTCAGTGTTTGTGTC	CATATCCTCCTCAGGCTTTC	189
LCAD	NM_007381.4	TCACCACACAGAATGGGAGA	ACGCTTGCTCTTCCCAAGTA	155
MCAD	NM_007382.5	TTGAGTTGACGGAACAGCAG	AGTTTGCACCCCTGTACACC	248
NRF1	NM_001164226.1	AAGTATTCCACAGGTCGGGG	TGGTGGCCTGAGTTTGTGTT	238
PGC1α	NM_008904.2	GGGCCAAACAGAGAGAGAGG	GTTTCGTCCGACCTGCGTAA	250
PPARα	NM_011144.6	GAGGGTTGAGCTCAGTCAGG	GGTCACCTACGAGTGGCATT	161
SIRT1	NM_019812.3	TACCCCATGAAGTGCCTCAA	AACCAATTCCTTTTGTGGGC	203
Tfam	NM_009360.4	GAGGCCAGTGTGAACCAGTG	GCTCTGAAGCACATGGTCAA	158
UCP2	NM_011671.5	GCGTTCTGGGTACCATCCTA	GCTCTGAGCCCTTGGTGTAG	156
UCP3	NM_009464.3	GAATCTCCGTTTTGAACAAG	ACGGAGGACTAAAACTCTCC	147
VLCAD	NM_017366.3	TATCTCTGCCCAGCGACTTT	TGGGTATGGGAACACCTGAT	175

CPT1α, carnitine palmitoyltransferase 1α; CPT1β, carnitine palmitoyltransferase 1β; CYP24, 1,25-dihydroxyvitamin D3 24-hydroxylase; CYP27, 25-Hydroxyvitamin D3 1-alpha-hydroxylase; LCAD, long-chain acyl-CoA dehydrogenase; MCAD, medium-chain acyl-CoA dehydrogenase; NRF1, nuclear respiratory factor 1; PGC1α, peroxisome proliferative activated receptor gamma coactivator 1α; PPARα, peroxisome proliferator-activated receptor α; SIRT1, sirtuin 1; Tfam, mitochondrial transcription factor A; VLCAD, very long-chain acyl-CoA dehydrogenase.

Table S2. Rat primers used for quantitative real-time polymerase chain reaction (qRT-PCR)

Gene	GeneBank no.	Forward sequence (5′-3′)	Reverse sequence (5′-3′)	Product size (bp)
β-actin	NM_031144.3	ACGGTCAGGTCATCACTATC	CAGGGCAGTAATCTCCTTCT	230
CPT1α	NM_031559.2	ATGACGGCTATGGTGTCTCC	GTGAGGCCAAACAAGGTGAT	154
CPT1β	NM_013200.1	TGTACTAGCGAGTCCACGGC	GGTGTTTTTCGGAGGCTTTC	100
CYP24	NM_201635.3	ACCTGGCTCTCTGCTGGATA	GCTCTGGTCCTTGAAGTTCG	239
CYP27	NM_053763.1	GTGCTTGACCTGACTCAGCA	AAGCATGGAAGGATCAGTGG	185
LCAD	NM_012819.1	CCTACAGCTGCATGAAACCA	GACGATCTGTCTTGCGATCA	229
NRF1	NM_001100708.1	AGATGCTAATGGCCCAGATG	AGCTCTGCCTGGTTGTTTGT	199
PGC1α	NM_031347.1	GACAAGACCAGTGAACTACG	CTCGACACGGAGAGTTAAAG	211
PPARα	NM_013196.1	TACCTGTGAACACGATCTGA	GCTAGTCTTTCCTGCGAGTA	136
SIRT1	NM_001372090.1	AGGGAACCTCTGCCTCATCT	GAGGTGTTGGTGGCAACTCT	199
Tfam	NM_031326.1	ATCAAGACTGTGCGTGCATC	AAAGCCCGGAAGGTTCTTAG	217
UCP2	NM_019354.3	ACTGTCGAAGCCTACAAGAC	CACCAGCTCAGTACAGTTGA	111
UCP3	NM_013167.2	CAGTGACCTGTGCTCAACCC	CCACAGTCCCCTGACTCCTT	146
VLCAD	NM_012891.2	GCATCTTGCTCTATGGCACA	ACTTTCCACAGGGGCTAGGT	156

CPT1α, carnitine palmitoyltransferase 1α; CPT1β, carnitine palmitoyltransferase 1β; CYP24, 1,25-dihydroxyvitamin D3 24-hydroxylase; CYP27, 25-Hydroxyvitamin D3 1-alpha-hydroxylase; LCAD, long-chain acyl-CoA dehydrogenase; NRF1, nuclear respiratory factor 1; PGC1α, peroxisome proliferative activated receptor gamma coactivator 1α; PPARα, peroxisome proliferator-activated receptor α; SIRT1, sirtuin 1; Tfam, mitochondrial transcription factor A; VLCAD, very long-chain acyl-CoA dehydrogenase.

nutrients

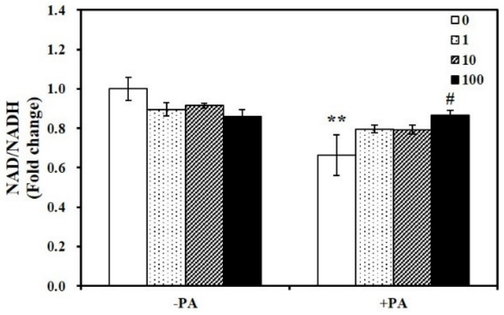

Figure S1. 1,25-dihydroxyvitamin D (1,25(OH)2D) increases the ratio of NAD to NADH. C2C12 muscle cells were pretreated with palmitic acid (PA, 0.5 mM, 24 h) and then incubated with 1,25(OH)2D (0, 1, 10, or 100 nM, 24 h). The NAD/NADH ratio was measured by a colorimetric assay (Abcam), normalized to their relative protein concentrations, and expressed as the fold change to vehicle control. Results are expressed as mean ± SEM from at least two independent experiments (n = 8 per group). ** $p < 0.01$ compared to vehicle control. # $p < 0.05$ compared to PA control.
